# Necrotizing granulomatous inflammation mimicking skeletal metastasis: a possible differential diagnosis

**DOI:** 10.1186/s41824-022-00151-4

**Published:** 2022-10-28

**Authors:** Deepanksha Datta, T. Ravichandran, Rajesh Kumar, Rashim Sharma, Deepak Vedant

**Affiliations:** 1 Department of Nuclear Medicine, All India Institute of Medical Sciences, Jodhpur, India; 2 Department of Pathology and Lab Medicine, All India Institute of Medical Sciences, Jodhpur, India

**Keywords:** Necrotizing granulomatous inflammation, Pott’s spine, Carcinoma cervix, F-18 FDG PET/CT

## Abstract

Tuberculosis is an endemic disease in India for decades, and its coexistence in the patients with malignancy cannot be ignored. The non-specific uptake of 2-deoxy-2-[fluorine-18] fluoro-d-glucose in active infection and malignancy can affect the diagnosis and management of patients. However, characteristic anatomical features of the lesion aid not only in its localization but also in diagnosis. We share an interesting case of necrotizing granulomatous inflammation of dorsal spine mimicking skeletal metastases in a treated case of carcinoma cervix.

## Introduction

An important indication of the 2-deoxy-2-[fluorine-18] fluoro-d-glucose positron emission tomography integrated with computed tomography (F-18 FDG PET/CT) is the localization and characterization of a pathological lesion suspicious of malignant potential. The major advantage of hybrid imaging is the concurrent availability of functional and anatomical details of the lesions. Due to non-specific uptake of F-18 FDG in active infection, inflammation and neoplastic process PET has limited specificity in differentiating infection from malignancy. However, the pattern and sites of FDG uptake along with diagnostic CT help in anatomical localization of the disease and also in characterization of the lesion based on typical radiological features. We highlight the utility of F-18 FDG PET/CT in the accurate diagnosis of Pott’s spine mimicking skeletal metastasis in a treated case of carcinoma cervix.

## Case report

**Fig. 1:**
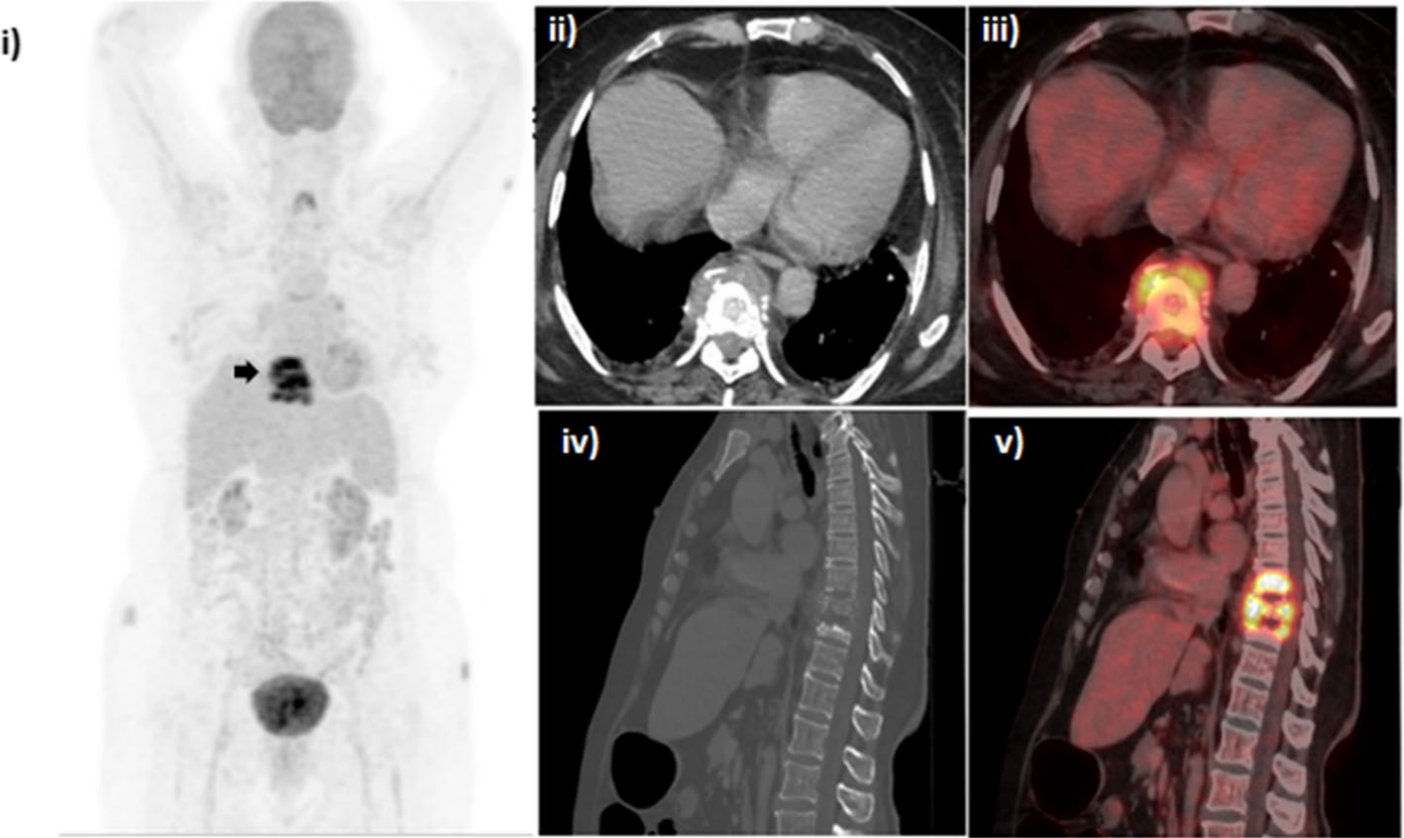
F-18 FDG PET/CT, **i** MIP images, **ii**–**v** CT and fused images. Black arrows show the pathological lesion

**Fig. 2:**
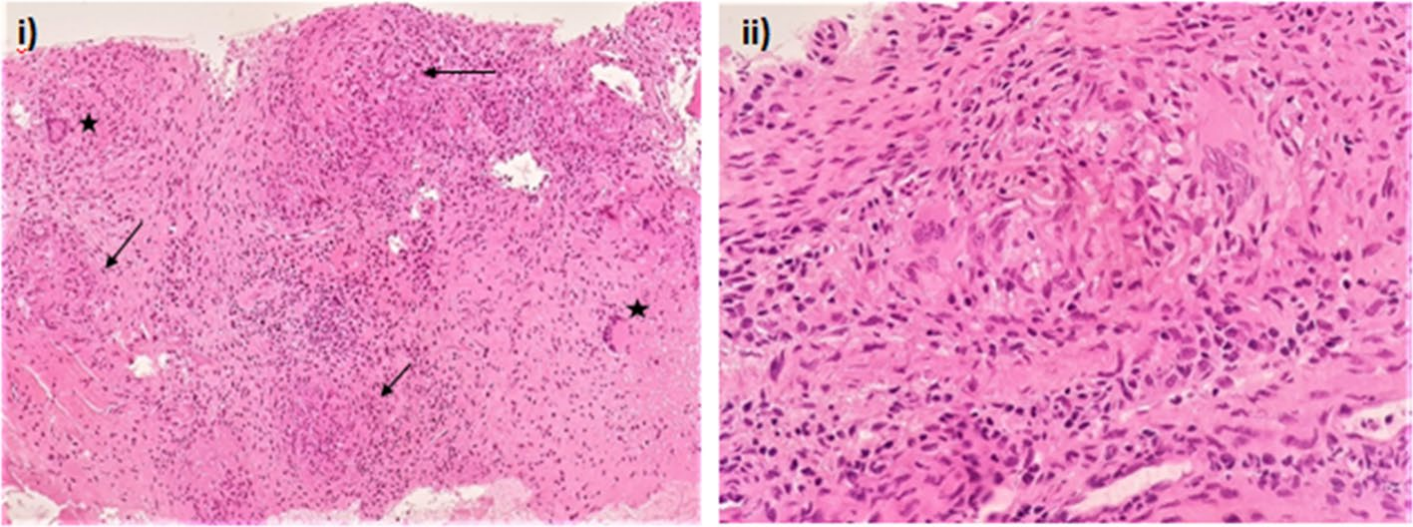
**i** Multiple necrotizing epithelioid cell granulomas (arrows) along with langerhans giant cells (black asterisks) (H and E, 20X) **ii** High power view of epithelioid cell granuloma along with giant cells (H and E, 40X)

A 77-year-old female was a known case of carcinoma cervix and completed chemo-radiotherapy 5 years ago (records unavailable) and had recent onset of bilateral lower limb weakness. F-18 FDG PET/CT (Fig. 1) showed hypermetabolic contiguous lytic lesions in D8-10 vertebrae with paradiscal cortical erosions, paravertebral soft tissue component, adjacent spinal canal extension and partial collapse of D9 vertebrae. This lesion was solitary, and there was no other imaging evidence of abnormality noted elsewhere, especially in pelvis, lung, liver and lymph nodes. Thus, loco-regional recurrence and distant metastases were ruled out. The radiological findings were typical for Pott’s spine. Paravertebral biopsy was advised and histopathology confirmed necrotizing granulomatous inflammation (Fig. 2).

## Discussion

Bone is the third most common distant metastases in cervical cancer after lungs and liver and can be found in any stage (Thanapprapasr et al. 2010; Hacker et al. 2005; Hage et al. 2000). The spine is the most common site of skeletal metastases (Thanapprapasr et al. 2010). According to an overview, up to 25% of tuberculosis cases are extra-pulmonary, and among them around 50% are Pott’s spines (Falagas et al. 2010). As per the Global Tuberculosis Report of WHO 2021, India is unfortunately among the 8 countries that account for the two-thirds of the global burden with high prevalence of multi-drug resistance and under-reporting of cases (Global Tuberculosis Report 2021). Tuberculosis and malignancy are risk factors for each other, and coexistence of tuberculosis in such cases cannot be ignored (Chen et al. 2021; Nanthanangkul et al. 2020). Also, the radiological appearances of tuberculosis and malignancy are very similar, thus increasing the confusion for diagnosis (Xiang et al. 2021). Pott’s spine is a known common differential for lytic skeletal metastases (Liu et al. 2017; Ye et al. 2016; Go et al. 2012), and non-specific uptake of F-18 FDG in active infection and inflammation reduces its specificity. The typical radiological features of Pott’s spine include anterior destruction of vertebral body, end plate erosions, loss of disc height, paravertebral masses and contiguous multi-vertebral involvement (Rivas-Garcia et al. 2013). However, the atypical findings are also increasingly observed like non-contiguous vertebral involvement and disc sparing (Go et al. 2012; Rivas-Garcia et al. 2013; Polley and Dunn 2009; Moore and Rafii 2001; Babhulkar et al. 1984; Yalniz et al. 2000**).** In this case, a latency of 5 years in emergence of symptoms with compressive neuropathy as the first symptom, and the solitary lesion with characteristic contiguous involvement of vertebrae and paradiscal lesions were supportive of granulomatous aetiology than metastases.

## Conclusion

Though FDG is not specific in differentiating infection from malignancy, it helps in localization of the active disease process. And typical appearance of the lesion on CT can aid in its characterization. Thus, the hybrid imaging with FDG PET/CT is preferred in characterization of a lesion, especially in equivocal or discordant lesions.

## Data Availability

Not applicable.
